# *dandelionR*: Single-cell immune repertoire trajectory analysis in R

**DOI:** 10.1016/j.csbj.2025.06.047

**Published:** 2025-06-30

**Authors:** Jiawei Yu, Xiaohan Xu, Nicholas Borcherding, Zewen Kelvin Tuong

**Affiliations:** a Ian Frazer Centre for Children’s Immunotherapy Research, Child Health Research Centre, Faculty of Health, Medicine and Behavioural Sciences, The University of Queensland, Australia; b Department of Pathology and Immunology, Washington University School of Medicine, Saint Louis, MO, USA

**Keywords:** Single-cell, TCR-seq, Trajectory analysis

## Abstract

Integration of single-cell RNA-sequencing (scRNA-seq) and adaptive immune receptor (AIR) sequencing (scVDJ-seq) is extremely powerful in studying lymphocyte development. A python-based package, *Dandelion*, introduced the VDJ-feature space method, which addresses the challenge of integrating single-cell AIR data with gene expression data and enhances trajectory analysis results. However, no R-based equivalent or similar methods currently exist. To fill this gap, we present *dandelionR*, an R implementation of *Dandelion*’s trajectory analysis workflow, bringing the VDJ feature space construction and trajectory analysis using diffusion maps and absorbing Markov chains to R, offering a new option for scRNA-seq and scVDJ-seq analysis to R users.

## 1 Introduction

During the development of T and B cells, variable (V), diversity (D) and joining (J) genes of adaptive immune receptors (AIRs) recombine stochastically, introducing variability in the joining region [8], [27]. This process, known as V(D)J recombination, plays a critical role in generating diversity of the AIR repertoire (AIRR) [6]. The diversity of AIRR is essential to adaptive immunity [18], but remains challenging for single-cell RNA sequencing (scRNA-seq) analysis [12]. This challenge necessitates the integration of scRNA-seq with AIR sequencing (scVDJ-seq) [13].

*Scirpy*[23], *Dandelion*[25], [20], and *scRepertoire*[29], [4] are widely used tools for conducting scVDJ-seq analysis. Among these, *Dandelion*'s Python-based innovative strategy of creating a VDJ feature space addressed some challenges in integrating AIR data within scRNA-seq, arising from the mixture of categorical and continuous data characteristics inherent to AIRR data. The feature space was leveraged to enable trajectory analysis informed by both the gene expression and VDJ data, which improved the prediction accuracy of trajectories from double-positive T cells to CD4/CD8 T cells, demonstrating significant potential for future applications [25]. This improvement is particularly notable because most existing trajectory analysis tools rely solely on gene expression matrices. While this strategy is effective in many contexts, they may fall short in modelling lymphocyte development, where VDJ recombination critically determines the receptor-antigen affinity, which in turn influences the development direction and the final cell fate. Integrating VDJ features with transcriptomics profiles enables trajectory analysis tools to account for both gene expression and VDJ usage, offering a more comprehensive view of lymphocyte differentiation. However, no comparable integration method currently exists in R, limiting R users’ ability to perform such comprehensive analyses of lymphocyte trajectories.

Here, we introduce *dandelionR*, an R-based scVDJ-seq trajectory analysis tool replicating the trajectory analysis workflow of *Dandelion*. *dandelionR* enables the construction of the VDJ feature space to perform trajectory analysis using diffusion maps and absorbing Markov chains, with seamless interaction with *scRepertoire*. The current version is available on GitHub and through Bioconductor, along with user documentation and additional resources. This tool addresses existing gaps in functionality among current tools, offering researchers a more convenient solution for analysing immune repertoires and single-cell sequencing data in R. By doing so, it facilitates a deeper exploration of lymphocyte development and its functional mechanisms.

## 2 Methods

For trajectory analysis, *Dandelion* requires cell pseudobulks, typically with *Milo* [7], to construct the pseudobulked VDJ feature space. The feature space is then used as the input for *Palantir* [19], a trajectory analysis tool which employs diffusion maps and absorbing Markov chains to infer trajectory. *Palantir* produces pseudotime values and probabilities of each pseudobulk, which *Dandelion* subsequently projects back to each cell.

*dandelionR* is developed and tested in R v4.4.1 and is available through Bioconductor (from release 3.21) and can interact with *scRepertoire* v2.2.1 onwards. As an R implementation of *Dandelion*, it aims to reproduce the preprocessing, feature-space-building and result-projecting functions of the original software.

The typical workflow of *dandelionR* proceeds as follows:

### 2.1 Input

*dandelionR* uses a *SingleCellExperiment* object already combined with vdj data, such as from reading with *scRepertoire* [29], [4], or processed using the python package *Dandelion* with *AnnData* and converted to *SingleCellExperiment*.

### 2.2 Preprocessing

This step includes filtering cells with non-productive immune receptors and ambiguous VDJ chain status, e.g., orphan/incomplete or multiple TCRs in one cell, retaining only cells with relevant or complete VDJ data. Then, the remaining VDJ contigs that express the highest UMI counts in a cell are extracted for downstream analyses. Depending on the data source, some preprocessing steps may not be necessary and can be skipped or modified. For example, when using *scRepertoire*-derived data, the user should set ‘*already.productive = TRUE’* to skip the productive filtering process, as the filtering has already been handled as part of the *scRepertoire*’s standard workflow. Additionally, there are many parameters that users can adjust according to their analysis requirements. For example, they can set ‘*allowed_chain_status = NULL*’ to skip checking whether a cell has relevant TCR chains and accept all contigs. This flexibility allows for a highly customizable preprocessing workflow as per user requirements. A default set of parameters has been defined based on the original *Dandelion* workflow to replicate the initial findings [25].

### 2.3 Pseudobulking and feature space constructing

Pseudobulking can be achieved through the *miloR* (v2.0.0) package [7]. Within each pseudobulk, *dandelionR* will tabulate the usage of each VDJ gene to create the VDJ feature space.

### 2.4 Trajectory analysis

Using the constructed VDJ feature space as input, we can utilise trajectory analysis tools to obtain pseudotime values and branching probabilities of each pseudobulk.

### 2.5 Projection

The calculated pseudotime values and probabilities of each pseudobulk are then projected back onto individual cells, generating the final trajectory analysis results.

### 2.6 Data

To evaluate the reproducibility of dandelionR, we converted the same data from *Dandelion*’s tutorial, into *SingleCellExperiment* format. The original data, derived from [25], represents a real-world single-cell-level map of immune systems development, and contains gene expression data with VDJ information. To analyse this dataset, *Dandelion* filtered out cells lacking TCR sequencing or belonging to the CD137 or MAIT-sorted populations. To illustrate the developmental trajectory of T cells, *Dandelion* further subsetted the dataset to retain only five cell types: DP(P), the double positive T cell undergoing active proliferation, DP(Q), the double positive T cell with limited proliferation and active VDJ recombination, ABT/ENTRY, the immature αβ T cell, CD4 +T, CD4 single positive T cell, and CD8 +T, CD8 single positive T cell.

## 3 Results

### 3.1 Replication of workflow before trajectory inference

**Fig. 1:**
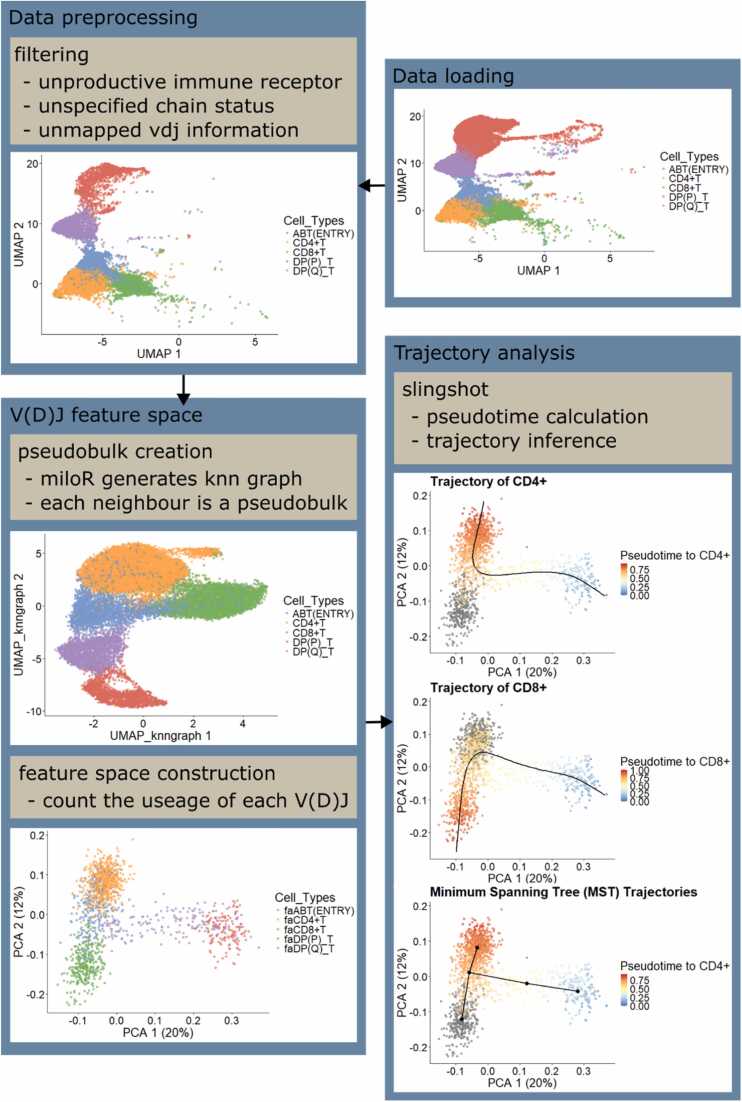
Overall workflow from preprocessing to trajectory analysis using *dandelionR*.

To replicate the *Dandelion* workflow before trajectory analysis (Fig. 1), we implemented the following functions:

The *dandelionR::setupVdjPseudobulk* function preprocessed the single-cell VDJ data by Suo et al. [24]. It then filtered out cells with non-productive or unclearly mapped alpha-beta TCR chains, extracting the main productive chain and storing it in a new column within the *colData* slot of the *SingleCellExperiment* object. Out of 65102 cells in the data, 17308 cells were retained after filtering due to having complete TCR information necessary for downstream analyses.

Using *MiloR* [7], we constructed a k-nearest neighbour graph from the preprocessed data, treating each neighbour as a pseudobulk. This step allocated cells to pseudobulks based on the similarity of their gene expression profiles. Subsequently, the *dandelionR::miloUmap* function utilised the graph’s adjacency matrix to generate a UMAP (Uniform Manifold Approximation and Projection). The *dandelionR::vdjPseudobulk* function then created a VDJ feature space by counting the usage of each gene in each pseudobulk. With 160 V/D/J genes and 1516 pseudobulks, the VDJ feature space captured features from both gene expression and VDJ information in a continuous data format.

### 3.2 Implementing trajectory inference based on absorbing Markov chains

The *Dandelion* workflow originally used the constructed feature space as an input for *Palantir*, a trajectory analysis tool, treating each pseudobulk as a cell and VDJ usage as gene expression information. *Palantir* employs probabilistic methods [19], which are primarily implemented in Python-based tools [9]. However, most R-based tools do not incorporate such methods. Instead, *TSCAN* [15], [14] utilises a self-developed travelling salesman problem (TSP) algorithm, *Slingshot* [22] combines both minimum spanning tree with a self-modified principal curve, and *destiny* [1] applies a diffusion map. While *Ouija* [5] is a probabilistic method utilising a Bayesian latent variable model, it is unsuitable for our dataset. This is because *Ouija* is limited to data with a linear topology, whereas we are certain that our dataset exhibits a bifurcation between CD4 + and CD8 + cells.

Since there are no direct *Palantir* equivalent or similar methods in R, we first attempted to use *Slingshot* [22] for downstream analysis, following the comparison framework provided by *dynverse* [17]. However, *Slingshot* does not provide outputs analogous to branching probabilities (see Supplementary Information 1). To address this limitation, we sought to implement *Palantir*’s trajectory analysis function in R. We anticipate that this approach could not only address the lack of branching probability in our workflow but also help fill a critical gap in the R community, where probabilistic methods for trajectory analysis remain scarce.

In the original *Dandelion* workflow, *Palantir* first identifies waypoints after preprocessing and then uses a diffusion map to compute diffusion pseudotime on each cell [19]. These waypoints are subsequently employed to construct an absorbing Markov chain, which calculates transition probabilities. Finally, pseudotime and branch probabilities derived from the waypoints are projected onto individual cells.

**Fig. 2:**
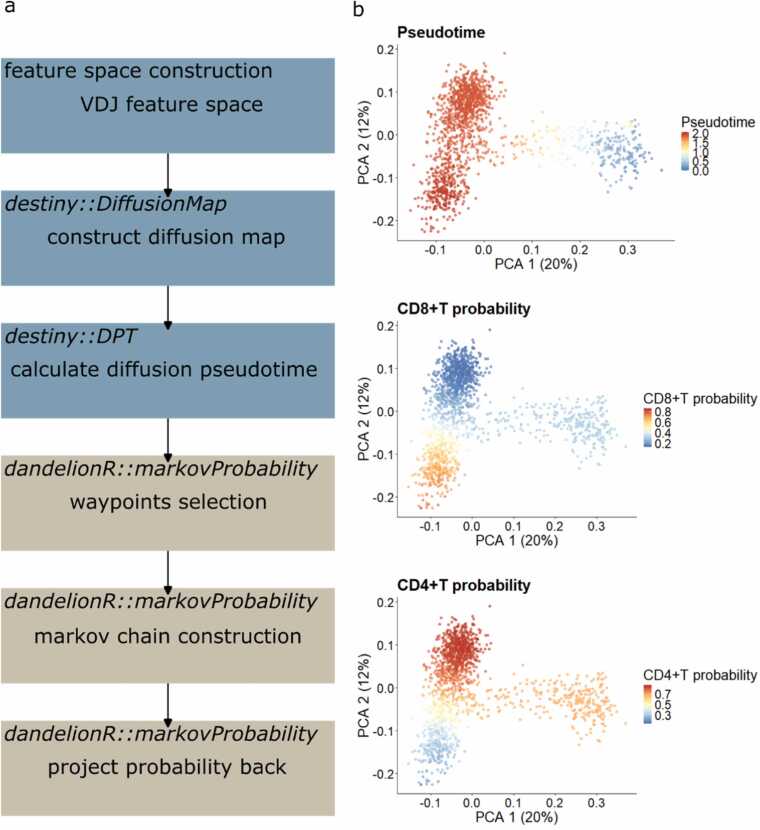
Trajectory analysis with Markov chain in *dandelionR*. (a) *dandelionR*’s trajectory analysis workflow that incorporates outputs from diffusion maps generated by *destiny*. (b) Pseudotime and branching probabilities of each pseudobulk after trajectory analysis.

We utilised the *destiny* package to calculate the diffusion map and pseudotime. Subsequent processes—including waypoint selection, absorbing Markov chain construction, probability calculation, and projection—were implemented independently and consolidated into a function called *dandelionR::markovProbability* (Fig. 2).

**Fig. 3:**
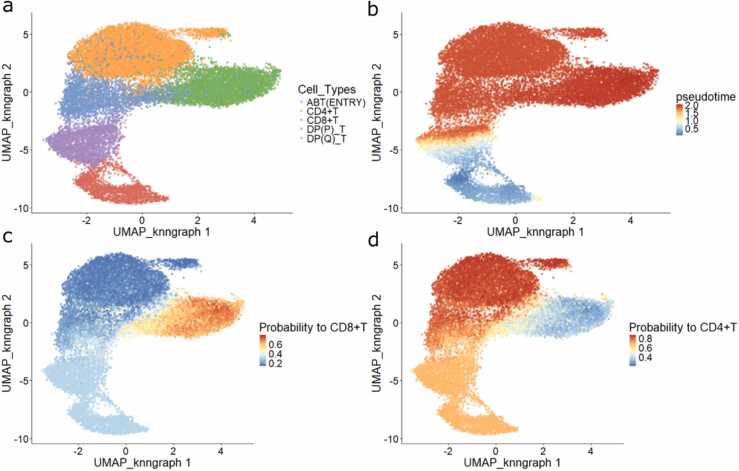
Projection of pseudobulked trajectory results to single cells. (a) Single-cell UMAP plot coloured by cell types. (b, c, d) UMAP coloured by pseudotime, branching probabilities to CD8 +T and CD4 +T of individual cells.

Finally, the probabilities and pseudotime of each pseudobulk computed by VDJ feature space were projected back to individual cells through the *dandelionR::projectPseudotimeToCell* function (Fig. 3). A total of 39 cells were removed due to not belonging to any neighbourhoods.

### 3.3 Benchmarking

To evaluate the computational performance of *dandelionR*, we compared the total runtime time and the peak memory usage of the tutorial workflows in both *dandelionR* and D*andelion*. The results are summarised in Supplementary Information.

To assess the agreement between *dandelionR* and the original D*andelion*, we calculated several statistical metrics: the Pearson correlation coefficient, the Lin's Concordance Correlation Coefficient (CCC), the Kendall rank correlation coefficient, and the mean absolute error (MAE). In addition, we generated Bland-Altman plots to visualise the difference between outputs of the two implementations.

#### 3.3.1 Benchmark 1: *dandelionR* (R) versus *Dandelion* (Python)

We first compared the outputs of the overall workflow. The *dandelionR* output includes 17,281 cells, while the original *Dandelion* output contains 17,234 cells, with 17,208 shared between the two. This discrepancy may stem from differences in random seed handling between R and Python, particularly during neighbour selection in the *miloR* step. For each intersecting cell, we compared both pseudotime and CD4 + branching probability between *dandelionR* and *Dandelion* (Fig. S1). As the dataset only has two terminal fates, the sum of CD4 +T probability and CD8 + probability from each cell equals one. Therefore, we report only the results of CD4 + probability.

The Pearson correlation coefficients are high for both pseudotime and CD4 + probability, indicating strong linear relationship between the two implementations. However, Lin's CCC for CD4 + probability (0.850) falls below the commonly accepted threshold of 0.9, suggesting room for improvement in agreement. Similarly, the Kendall rank correlation for pseudotime (0.684) indicates only moderate consistency in rank ordering. Moreover, the MAE values reflect a degree of bias between the outputs. We next explored which steps in the workflow contributed to these differences.

#### 3.3.2 Benchmark 2: using *Palantir*’s diffusion map and pseudotime in both workflows

The absorbing Markov chain in *dandelionR* was implemented via self-developed functions, which allows flexible modification to improve the agreement between workflows. Moreover, as it and the projection step constitute the final stage of the pipeline, its performance can be assessed in isolation. These factors motivated us to examine the effect of this step first.

To evaluate the agreement of the absorbing Markov chain step, we used *dandelionR* to construct the VDJ feature space (Fig. S2). The resulting data were converted to *AnnData* format to serve as input for *Palantir*. The eigenvectors and eigenvalues of *Palantir*’s diffusion map were saved and transferred back to R to reconstruct the *DiffusionMap* object by *destiny* package. Additionally, the pseudobulk-level pseudotime computed by *Palantir* was also transferred back to R. The reconstructed *DiffusionMap* object and the pseudotime were used as the inputs of absorbing Markov chain of *dandelionR*. The resulting outputs were compared with those from *Palantir*, with both workflows using the same input feature space. Since both *dandelionR* and *Dandelion* use the same pseudobulk-level pseudotime, the possible discrepancy in final pseudotime could only originate from the projection step after absorbing Markov chain. With all three correlation coefficients equal to 1.000 and a negligible MAE (2.137 ×10⁻⁸), we confirmed that no difference arose in the projection step.

The remaining discrepancy observed in CD4 + probability therefore should have stemmed from differences in the absorbing Markov chain implementation itself. By using an identical diffusion map representation and pseudotime as input, we observed the improved agreement in all metrics except for MAE. Notably, the Kenall increased from 0.790 to 0.925, suggesting that ranking consistency of the branching probability is highly sensitive to differences in the diffusion map and pseudotime. Lin’s CCC also increased, but still remained below 0.9, indicating room for further improvement.

#### 3.3.3 Benchmark 3: modifications in absorbing Markov chain implementation

After examining the exact procedures in *Palantir*’s and *dandelionR*’s absorbing Markov chain implementations, we hypothesized that the primary source of discrepancy lies in the K-nearest neighborhood (KNN) graph construction step within the Markov chain building process.

*Palantir* constructs the KNN graph using *scikit-learn*’s *NearestNeighbors* function, which applies K-dimensional (KD)-tree methods when the dataset is small. In contrast, *dandelionR* uses the *makeKNNGraph* function from the *bluster* package in Bioconductor, which relies on brute-force approach for small datasets.

To test this hypothesis, we substituted *bluster* with *RANN*, an R package which also employs KD-tree for KNN graph construction (Fig. S3). We observed substantial improvements in Pearson correlation, Lin’s CCC, and MAE for CD4 + probability. While the Kendall rank correlation is slightly lower than that in Benchmark 2 (0.925–0.895), it still indicates a high degree of ranking agreement and is considered acceptable.

Based on the improved benchmarking performance, we have elected to switch to RANN (from *bluster*) to be the default method for KNN construction in *dandelionR*’s function for calculating branch probabilities.

#### 3.3.4 Benchmark 4: compatibility of diffusion map representations

Finally, we examined the agreement between *destiny* and *Palantir* in the diffusion map and pseudotime calculation step. Using *dandelionR*, we first constructed the VDJ feature space. This time, however, we used the eigenvectors and eigenvalues produced by *destiny* as the input to *Palantir* (Fig. S4).

Nevertheless, the pseudotime produced by *Palantir* under this setting is problematic. For example, DP(Q) cells are assigned the highest pseudotime value, which contradicts the expected developmental progression. As a result, all metrics are poor. This result indicates that the diffusion map representation produced by *destiny* is not compatible with *Palantir*’s framework.

However, the CD4 + fate probabilities, although less consistent to previous benchmarks, still retain a biological meaningful pattern and show relatively high Pearson correlation (0.959). This suggests that the absorbing Markov chain is somewhat robust to suboptimal pseudotime inputs, and that the *destiny* diffusion map still captures relevant biological structure in the feature space.

### 3.4 Integration with scRepertoire workflow

*scRepertoire* integrates VDJ information with gene expression, storing it in the *colData* of a *SingleCellExperiment* object. The VDJ usage we need lies in the column *‘CTgene’*, where V, D, J genes are separated by periods and TRA and TRB chains are separated by an underscore. For instance, the entry *‘TRAV23.TRAJ21.TRAC_TRBV5–1.NA.TRBJ2–1.TRBC2’* indicates that the TRA chain has V gene TRAV23 and J gene TRAJ21, while TRB chain contains V gene TRBV5–1, unclear D gene, and J gene TRBJ2–1. If any of the chains is absent, it is represented as *‘NA’*, such as *‘TRAV13–2.TRAJ23.TRAC_NA’*, where the TRB chain is absent.

However, the calculation of VDJ feature space treats each V, D, and J gene separately. To address this, we developed an internal function that extracts the VDJ information from the *‘CTgene’* column generated by *scRepertoire*, splitting the V, D and J genes and storing them in individual columns. This function was incorporated in *dandelionR::setupVdjPseudobulk* to ensure compatibility with *scRepertoire*. Additionally, we introduced parameters to allow users to skip the additional filtering, as it is already performed within *scRepertoire*. To prevent errors from splitting the V, D, J genes correctly, users should first ensure that cells with multiple contigs have already been filtered by setting *‘filterMulti = TRUE’* during *scRepertoire::combineTCR* step.

**Fig. 4:**
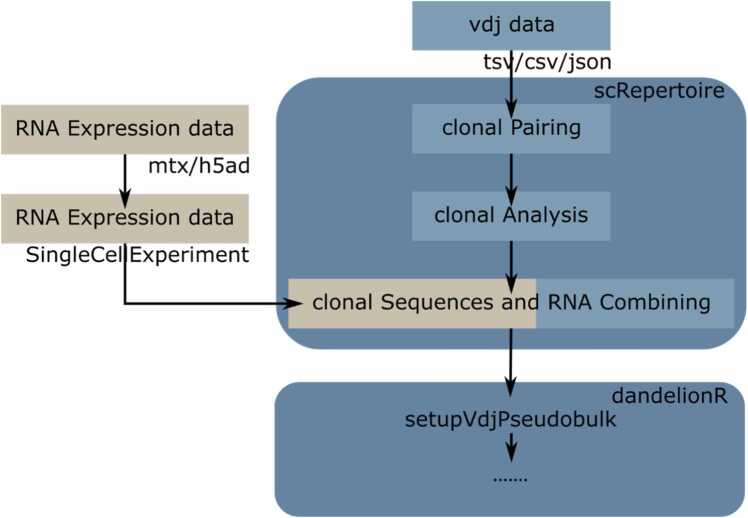
*dandelionR*’s integration with *scRepertoire*’s workflow. Users would perform preprocessing of the scVDJ-seq data as per standard *scRepertoire* workflow and this can then serve as input for *dandelionR*.

Overall, the implementations above allowed *dandelionR* to function as a downstream tool for *scRepertoire* (Fig. 4), enabling further trajectory analysis after the combination of VDJ information with gene expression data.

## 4 Discussion

We have successfully reproduced *Dandelion*’s trajectory analysis workflow, and incorporated it to *scRepertoire*’s workflow. These steps enable *dandelionR* to function as an R-based trajectory analysis tool that utilised both gene expression data and VDJ information combined by *scRepertoire*. The consistency between the outputs of *dandelionR* and the original *Dandelion* was evaluated through four benchmarking analyses. These comparisons include assessments of pseudotime and branching probability using multiple statistical metrics.

*dandelionR* implements part of *Palantir*’s trajectory analysis functions based on absorbing Markov chain. Originally, absorbing Markov chain, with the ability to define terminal states from the markov chain in an unsupervised step, allows the *Palantir* package to handle data with multifurcation topology. We have implemented the ability to define terminal states as well. However, the package we used to perform diffusion map, *destiny*, is only suitable for a dataset with a bifurcation or linear topology, which limits the versatility of *dandelionR*’s trajectory inference function on more complex developmental structures.

To overcome this limitation, users may choose to substitute the default trajectory inference step with other R-based tools such as *Slingshot*, which supports tree-like structures, although *Slingshot* does not produce branching probability (as discussed in Supplementary Information 1). Pseudotime values derived from such tools at the pseudobulk level can be projected back to single-cell resolution using the *dandelionR::projectPseudotimeToCell* function.

Moreover, *dandelionR* has not yet been applied to B cell development data, which involves more complex processes than T cell development. For instance, after V(D)J recombination, if an immature B cell is self-active, it may re-upregulate RAG to undergo additional rearrangements, a process known as receptor editing [11], [16], [26]. This forms a cyclic trajectory, which cannot be represented by absorbing Markov chains, as these inherently model unidirectional progression toward absorbing (terminal) states.

Adapting trajectory inference methods to account for cyclic developmental processes is non-trivial, requiring both algorithmic changes and appropriate datasets. Furthermore, the performance and assumptions of absorbing Markov chains in B cell development merit further investigation. In our search for suitable B cell datasets with paired BCR sequencing, we considered several published datasets. However, many did not meet key criteria: (1) derived from bone marrow, the site of B cell maturation; (2) sufficient cell numbers to capture intermediate developmental states; (3) availability of BCR sequencing to construct V(D)J feature space. For example, the Dong et al. dataset [10] could not be accessed due to restricted data access conditions and errors with the provided alternative accession number. The dataset from Tonglin et al., [28], although it includes healthy controls, only contained 972 healthy B cells, which is likely insufficient even for *miloR* neighbourhood construction. Two other papers e.g. Bandyopadhyay et al., [3] and Baccin et al., [2] contain suitable cell types and annotations but lack BCR sequencing. Finally, the paper from Strati and colleagues [21] focuses on immunotherapy of large B-cell lymphoma, making it less ideal for modeling normal development.

We also highlight the fundamental discrepancies in diffusion map construction and KNN graph construction between R and Python packages generally, which hinder our ability to fully implement the original *Dandelion* and *Palantir* workflow for TCR trajectory inference. In particular, for KNN graph construction, adopting a KD-tree-based approach yields results more consistent with the original *Dandelion* implementation. However, it remains unclear whether brute-force or KD-tree methods more accurately capture the underlying biological structure. Despite this ambiguity, our benchmarking demonstrates that the approaches are highly concordant in terms of global correlation, although subtle differences in cell trajectory ranking may persist. Based on the aforementioned limitations, future directions will focus on replacing *destiny* with alternative methods for conducting diffusion maps, which may improve interoperability and better capture underlying biological structures in the single-cell data.

Overall, while we have now developed a comparable TCR trajectory inference workflow compatible with R environments, available as a package through Bioconductor, we still recommend that users compare results against multiple trajectory inference tools, and where possible, across programming languages/environments, to assess the robustness of the inferred trajectories with respect to biological interpretation. Future work will explore implementing additional functionalities from the original *Dandelion* Python package e.g. filtering and network generation, as well as any future new functionalities, where possible.

## Author contributions

ZKT conceived the project. JY, NB and ZKT wrote the code. JY, and XX performed data analysis. JY and ZKT wrote and edited the manuscript. ZKT supervised the work.

## CRediT authorship contribution statement

**Nicholas Borcherding:** Writing – review & editing, Software. **Zewen Kelvin Tuong:** Writing – review & editing, Supervision, Software, Methodology, Conceptualization. **Jiawei Yu:** Writing – review & editing, Writing – original draft, Visualization, Software, Methodology, Investigation, Formal analysis, Data curation. **Xiaohan Xu:** Writing – review & editing, Data curation.

## Declaration of Competing Interest

The authors declare the following financial interests/personal relationships which may be considered as potential competing interests: N.B. was previously employed by Santa Ana Bio, Inc and Omniscope, Inc. The remaining authors have no conflicts of interest to declare.

## References

[1] Angerer P., Haghverdi L., Büttner M., Theis F.J., Marr C., Buettner F. (2016). destiny: diffusion maps for large-scale single-cell data in R. Bioinforma (Oxf Engl).

[2] Baccin C., Al-Sabah J., Velten L., Helbling P.M., Grünschläger F., Hernández-Malmierca P., Nombela-Arrieta C., Steinmetz L.M., Trumpp A., Haas S. (2020). Combined single-cell and spatial transcriptomics reveal the molecular, cellular and spatial bone marrow niche organization. Nat Cell Biol.

[3] Bandyopadhyay S., Duffy M.P., Ahn K.J., Sussman J.H., Pang M., Smith D., Duncan G., Zhang I., Huang J., Lin Y., Xiong B., Imtiaz T., Chen C.-H., Thadi A., Chen C., Xu J., Reichart M., Martinez Z., Diorio C., Tan K. (2024). Mapping the cellular biogeography of human bone marrow niches using single-cell transcriptomics and proteomic imaging. Cell.

[4] Borcherding N., Bormann N.L., Kraus G. (2020). scRepertoire: an R-based toolkit for single-cell immune receptor analysis. F1000Research.

[5] Campbell K.R., Yau C. (2019). A descriptive marker gene approach to single-cell pseudotime inference. Bioinforma (Oxf Engl).

[6] Carmona L.M., Schatz D.G. (2017). New insights into the evolutionary origins of the recombination-activating gene proteins and V(D)J recombination. FEBS J.

[7] Dann E., Henderson N.C., Teichmann S.A., Morgan M.D., Marioni J.C. (2022). Differential abundance testing on single-cell data using k-nearest neighbor graphs. Nat Biotechnol.

[8] Davis M.M., Bjorkman P.J. (1988). T-cell antigen receptor genes and T-cell recognition. Nature.

[9] Deconinck L., Cannoodt R., Saelens W., Deplancke B., Saeys Y. (2021). Recent advances in trajectory inference from single-cell omics data. Curr Opin Systems Biology.

[10] Dong C., Guo Y., Chen Z., Li T., Ji J., Sun C., Li J., Cao H., Xia Y., Xue Z., Gu X., Liang Q., Zhao R., Fu T., Ma J., Jiang S., Wu C., Fu Q., Guo G., Gu Z. (2024). Single-cell profiling of bone marrow B cells and early B cell developmental disorders associated with systemic lupus erythematosus. Arthritis Rheumatol.

[11] Gay D., Saunders T., Camper S., Weigert M. (1993). Receptor editing: an approach by autoreactive B cells to escape tolerance. J Exp Med.

[12] Heumos L., Schaar A.C., Lance C., Litinetskaya A., Drost F., Zappia L., Lücken M.D., Strobl D.C., Henao J., Curion F. (2023). Best practices for single-cell analysis across modalities. Nat Rev Genet.

[13] Irac S.E., Soon M.S.F., Borcherding N., Tuong Z.K. (2024). Single-cell immune repertoire analysis. Nat Methods.

[14] Ji Z., Ji H. (2016). TSCAN: Pseudo-time reconstruction and evaluation in single-cell RNA-seq analysis. Nucleic Acids Res.

[15] Ji Z., Ji H. (2019). Pseudotime reconstruction using TSCAN. Methods Mol Biol.

[16] Okoreeh M.K., Kennedy D.E., Emmanuel A.O., Veselits M., Moshin A., Ladd R.H., Erickson S., McLean K.C., Madrigal B., Nemazee D., Maienschein-Cline M., Mandal M., Clark M.R. (2022). Asymmetrical forward and reverse developmental trajectories determine molecular programs of B cell antigen receptor editing. Sci Immunol.

[17] Saelens W., Cannoodt R., Todorov H., Saeys Y. (2019). A comparison of single-cell trajectory inference methods. Nat Biotechnol.

[18] Scott J.K., Breden F. (2020). The adaptive immune receptor repertoire community as a model for FAIR stewardship of big immunology data. Curr Opin Syst Biol.

[19] Setty M., Kiseliovas V., Levine J., Gayoso A., Mazutis L., Pe’er D. (2019). Characterization of cell fate probabilities in single-cell data with Palantir. Nat Biotechnol.

[20] Stephenson E., Reynolds G., Botting R.A., Calero-Nieto F.J., Morgan M.D., Tuong Z.K., Bach K., Sungnak W., Worlock K.B., Yoshida M., Kumasaka N., Kania K., Engelbert J., Olabi B., Spegarova J.S., Wilson N.K., Mende N., Jardine L., Gardner L.C.S., Haniffa M. (2021). Single-cell multi-omics analysis of the immune response in COVID-19. Nat Med.

[21] Strati P., Li X., Deng Q., Marques-Piubelli M.L., Henderson J., Watson G., Deaton L., Cain T., Yang H., Ravanmehr V., Fayad L.E., Iyer S.P., Nastoupil L.J., Hagemeister F.B., Parra E.R., Saini N., Takahashi K., Fowler N.H., Westin J.R., Green M.R. (2023). Prolonged cytopenia following CD19 CAR T cell therapy is linked with bone marrow infiltration of clonally expanded IFNγ-expressing CD8 T cells. Cell Rep Med.

[22] Street K., Risso D., Fletcher R.B., Das D., Ngai J., Yosef N., Purdom E., Dudoit S. (2018). Slingshot: cell lineage and pseudotime inference for single-cell transcriptomics. BMC Genom.

[23] Sturm G., Szabo T., Fotakis G., Haider M., Rieder D., Trajanoski Z., Finotello F. (2020). Scirpy: a Scanpy extension for analyzing single-cell T-cell receptor-sequencing data. Bioinformatics.

[24] Suo C., Polanski K., Dann E., Lindeboom R.G.H., Vilarrasa-Blasi R., Vento-Tormo R., Haniffa M., Meyer K.B., Dratva L.M., Tuong Z.K., Clatworthy M.R., Teichmann S.A. (2024). Dandelion uses the single-cell adaptive immune receptor repertoire to explore lymphocyte developmental origins. Nat Biotechnol.

[25] Suo C., Polanski K., Dann E., Lindeboom R.G.H., Vilarrasa-Blasi R., Vento-Tormo R., Haniffa M., Meyer K.B., Dratva L.M., Tuong Z.K., Clatworthy M.R., Teichmann S.A. (2024). Dandelion uses the single-cell adaptive immune receptor repertoire to explore lymphocyte developmental origins. Nat Biotechnol.

[26] Tiegs S.L., Russell D.M., Nemazee D. (1993). Receptor editing in self-reactive bone marrow B cells. J Exp Med.

[27] Tonegawa S. (1983). Somatic generation of antibody diversity. Nature.

[28] Tonglin H., Yanna Z., Xiaoling Y., Ruilan G., Liming Y. (2021). Single-cell RNA-seq of bone marrow cells in aplastic anemia. Front Genet.

[29] Yang Q., Safina K.R., Borcherding N. (2024). ScRepertoire 2: enhanced and efficient toolkit for single-cell immune profiling. bioRxiv.

