# Supplementary material for: dandelionR: Single-cell immune repertoire trajectory analysis in R
Source: Comput Struct Biotechnol J. 2025 Jun 30;27:2890–7. doi: 10.1016/j.csbj.2025.06.047 (PMC12270724; doi:10.1016/j.csbj.2025.06.047)
Supplement: Supplementary file 1 — Supplementary material [file mmc1.docx]

**Supplementary Information and Appendices**

## **Supplementary Figures**


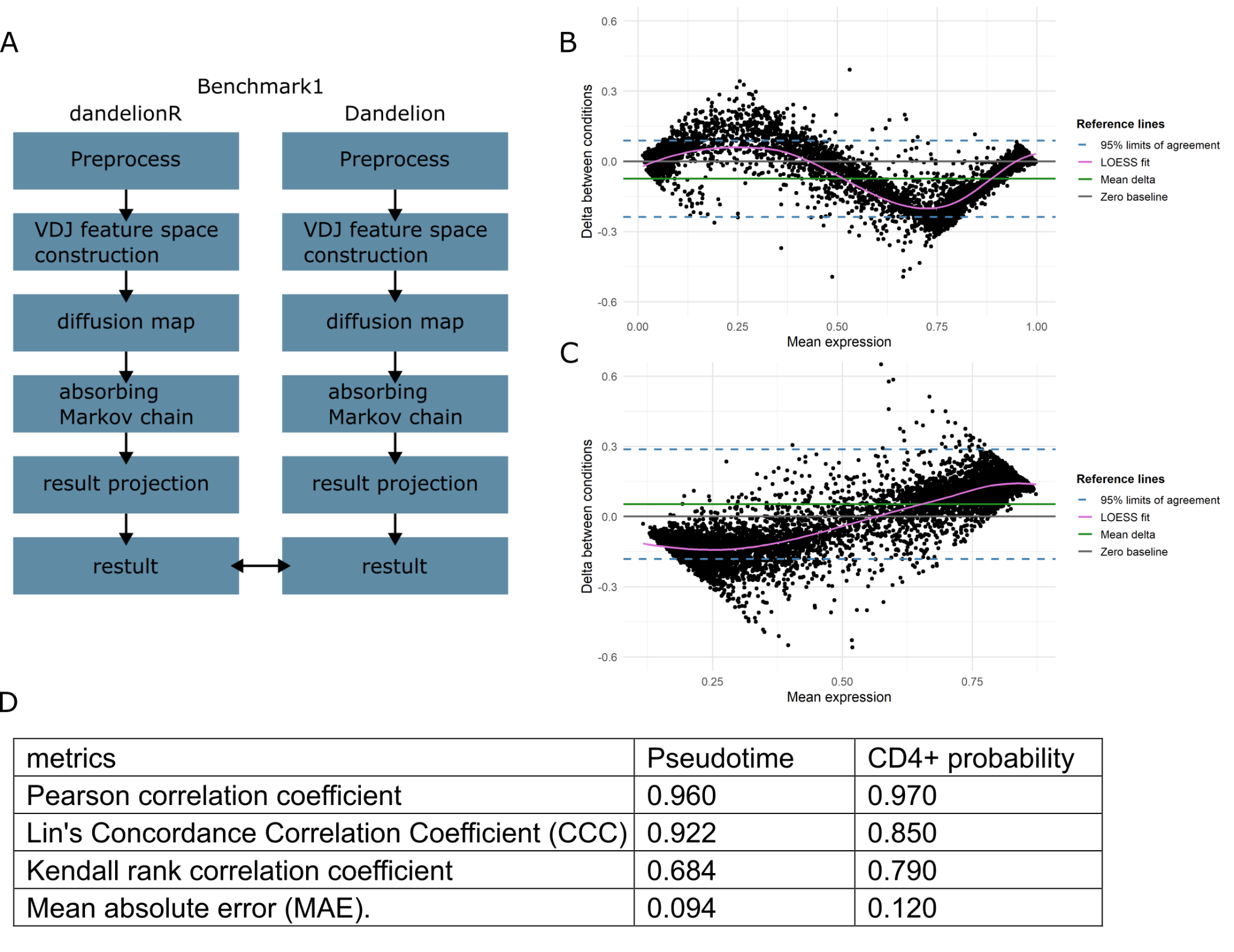


**Fig.S1 Workflow and Bland-Altman plot of benchmark 1.** Benchmark 1 compares the outputs of the *dandelionR* and *Dandelion* workflows using the dataset. (A) the workflow in the benchmark 1 (B) Bland-Altman plot of the pseudotime values. (C) Bland-Altman plot of CD4+ branching probability. Both (B) and (C) reveal systematic biases between the two implementations. (D) Summary of the four evaluation metrics used to assess the agreement between *Dandelion* and *dandelionR.*

## **
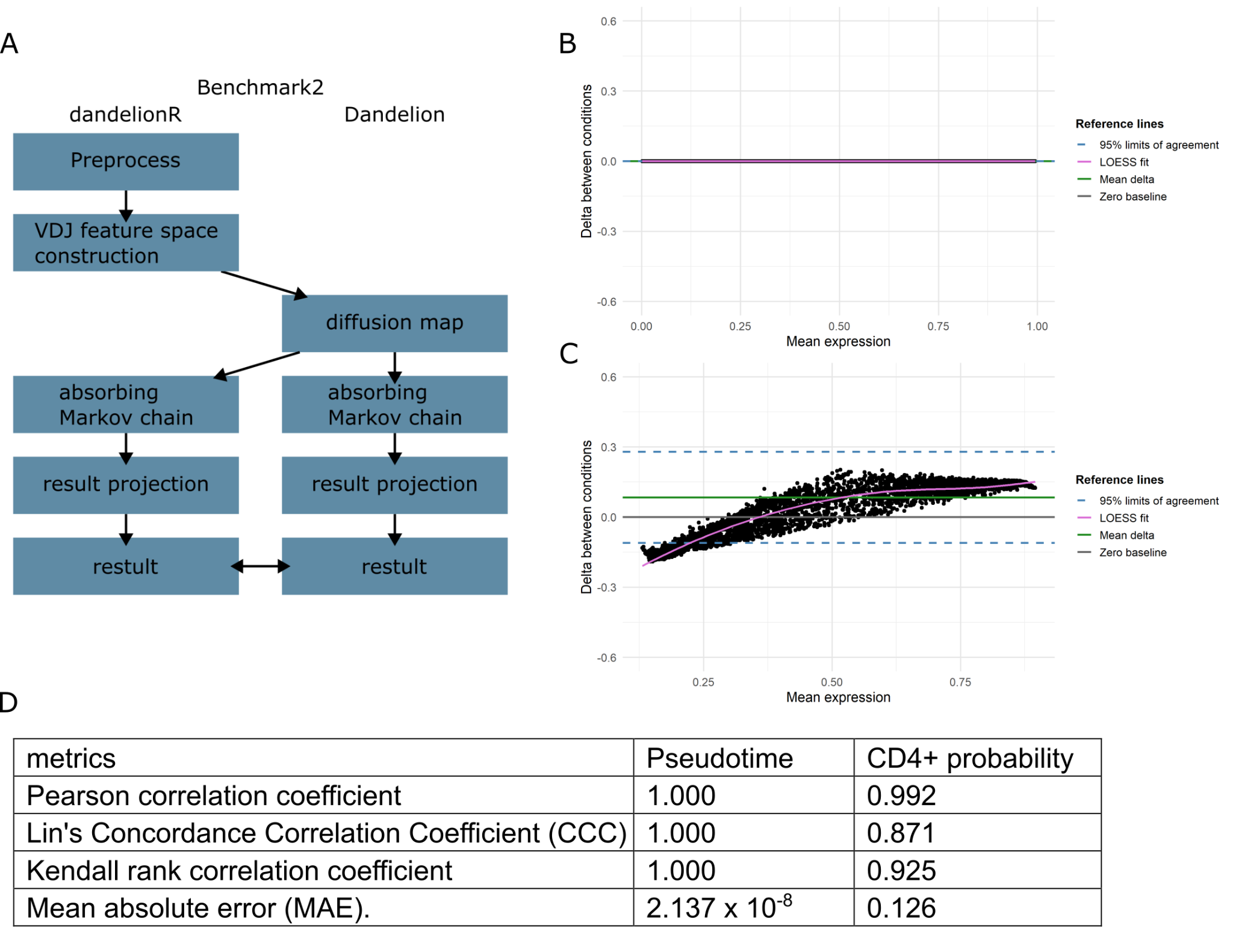
**

**Fig. S2 Workflow and Bland-Altman plot of benchmark 2.** Benchmark 2 evaluates the performance of absorbing Markov chains. (A) the workflows in the benchmark 2 share the same VDJ feature space, diffusion map representations and pseudotime, thereby eliminating potential discrepancies introduced by stochastic variation or diffusion map differences. (B) Bland-Altman plot of the pseudotime values. Data point, mean delta, zero line are nearly overlapping, indicating excellent agreement. This confirms that the projection step introduces negligible bias when identical pseudotime values are used. (C) Bland-Altman plot of CD4+ branching probability. A systematic bias is still present, but fewer points fall outside the 95% limits of agreement than in Fig. S1C.(D) Summary of the four evaluation metrics.
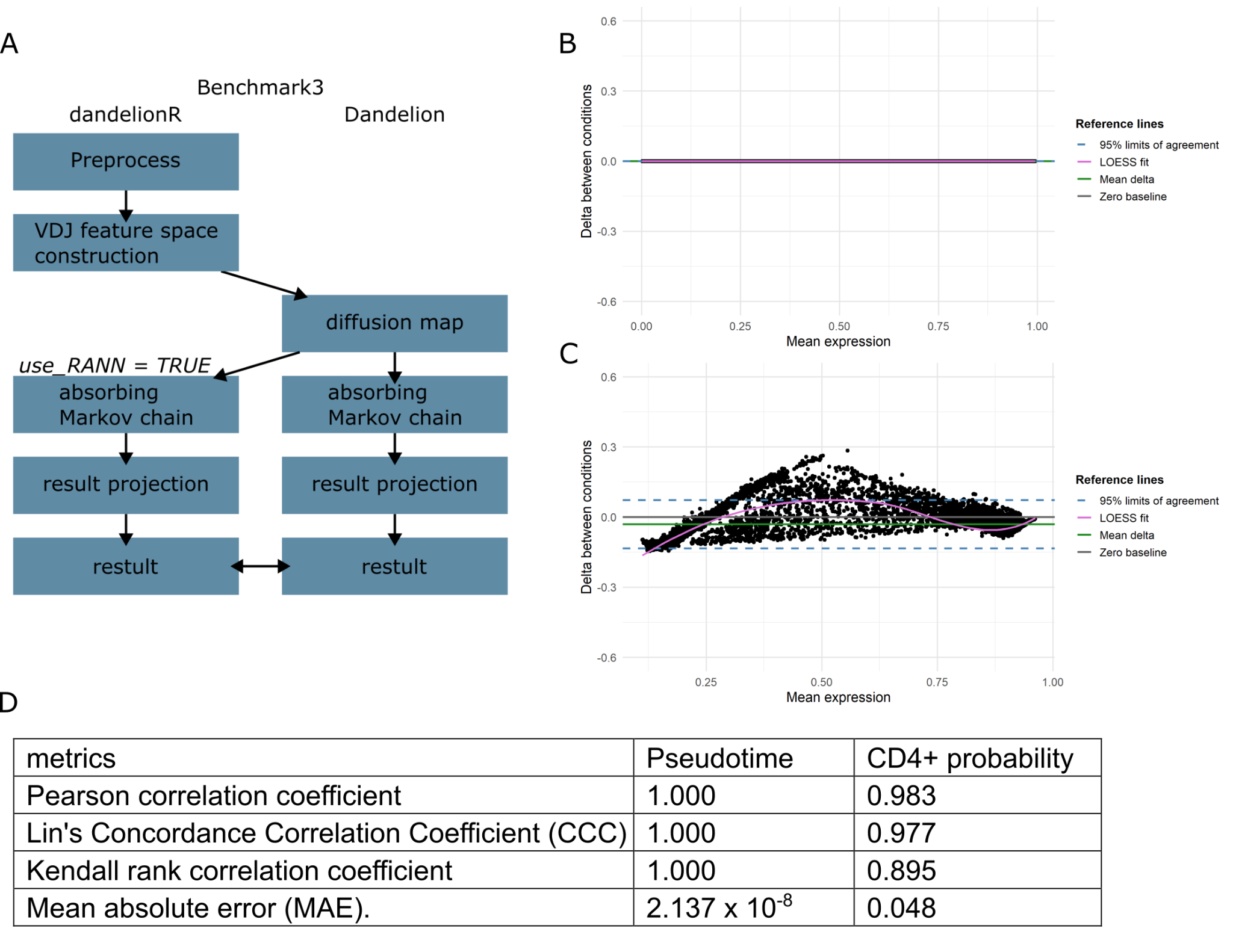


## **Fig. S3 Workflow and Bland-Altman plot of benchmark 3.** Benchmark 3 evaluates the performance of the absorbing Markov chain after replacing the KNN graph construction method from bluster with RANN. (A) the workflows in the benchmark 3 share the same VDJ feature space, diffusion map representations and pseudotime, as in Benchmark 2. The only difference is that Benchmark 3 employs *RANN* for KNN graph construction. (B) Bland-Altman plot of the pseudotime values. Data point, mean delta, zero line are nearly overlapping. (C) Bland-Altman plot of CD4+ branching probability. A systematic bias is still present; however, the mean delta line lies closer to the zero baseline, and the data points are more symmetrically distributed around zero, suggesting improved agreement compared to Benchmark 2. (D) Summary of the four evaluation metrics. Notable improvements are observed in Lin’s concordance correlation coefficient and mean absolute error (MAE), with only minor reductions in Kendall’s rank correlation coefficient and Pearson’s correlation coefficient.

##


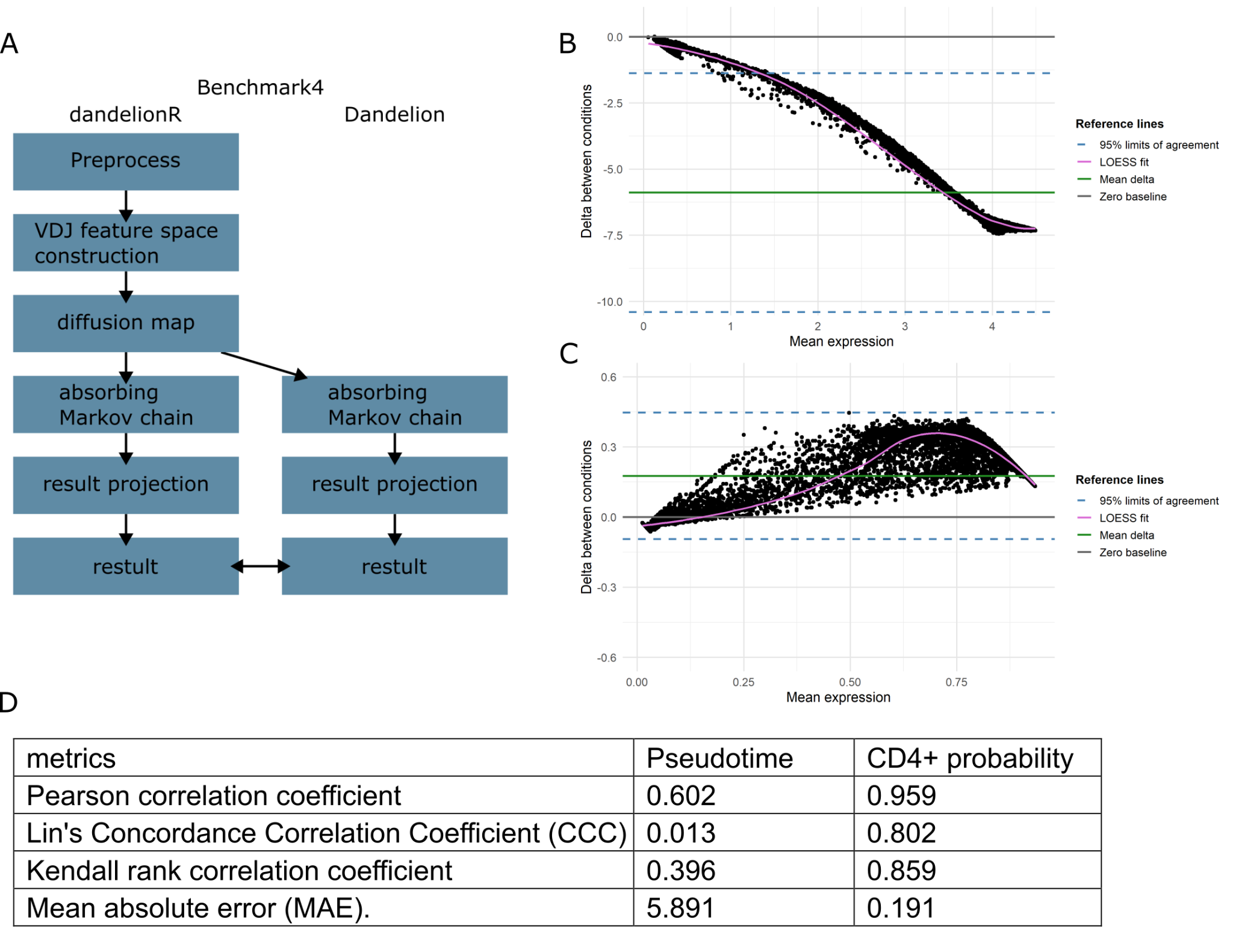


## **Fig. S4 Workflow and Bland-Altman plot of benchmark 4.** Benchmark 4 evaluates the compatibility of *destiny*’s diffusion map representations with *Palantir*’s workflow. (A) the workflows in the benchmark 4 share the same VDJ feature space. (B) Bland-Altman plot of the pseudotime values. The maximum delta exceeds 7.5, substantially larger than in other benchmarks, indicating very poor agreement between pseudotime values derived from *destiny* and *Palantir* diffusion maps. (C) Bland-Altman plot of CD4+ branching probability. A systematic bias is present, but the deviation is less extreme than that observed in (B), suggesting that the absorbing Markov chain retains partial robustness even under incompatible pseudotime input. (D) Summary of the four evaluation metrics.

## **Supplementary Information 1: experimental approach in selecting trajectory analysis tool**

We first used the trajectory analysis tools evaluation tool from *dynverse* (Saelens et al., 2019) to identify a suitable trajectory analysis tool for *dandelionR*. We ran the function *dynguidelines::guidelines_shiny()*, setting the number of cells to 1516, the number of genes to 160, and the expected topology to bifurcation. Among all the evaluated tools, *Slingshot* stood out with top accuracy, followed by four Python-based tools.

We first attempted trajectory inference with *Slingshot* to obtain pseudotime and trajectory results (Street et al., 2018). *Slingshot* modifies the principal curve in two ways, one of which is incorporated with cell weights. This helps assign cells to lineages. Additionally, the common point of origin and weight function ensure that pseudotime values remain close before two lineages bifurcate. The author also recommended using weight to identify lineage-specific genes. These features suggest its potential to model cell fate.

To explore whether “cell weights” can serve as a substitute for branching probabilities in *Palantir*, we plotted pseudobulks coloured by weight (**Supplementary Information Fig. 1**). Notably, cells located before a divergence typically receive weights close to 1 for both trajectories, indicating these cells should be assigned and have their pseudotime calculated within the lineage. However, for branching probability, we expected a value around 0.5, suggesting that the cell will differentiate to either a CD4+ T cell or a CD8+ T cell with similar probability. Additionally, the probability of differentiation into CD4⁺ T cells could be slightly higher, as CD4⁺ T cells are more abundant in an individual.
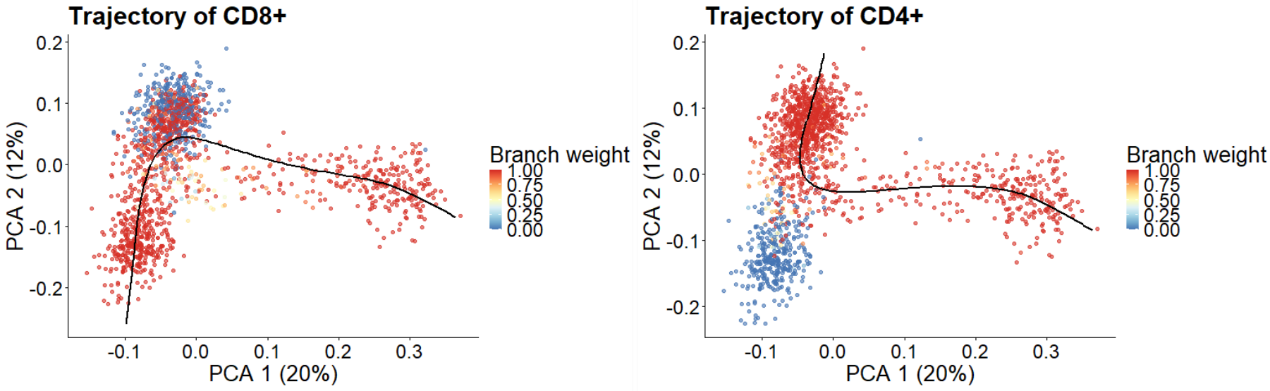
 **Supplementary Information Fig. 1. Weights produced by *Slingshot*.** Each dot represents a single pseudobulk coloured according to the weight values assigned by *Slingshot* within each lineage curve. The colour palette scales from 0 (blue) to 1 (red), where 0 indicates that the data point does not belong to the respective lineage.

## **Supplementary Information 2: Comparison on runtime and memory usage**

To evaluate the computational performance of *dandelionR*, we compared the total runtime and the peak memory usage of the tutorial workflows in both *dandelionR* and *Dandelion* **(Supplementary Information Table 1)**.

**Supplementary Information Table 1. Overall runtime and memory usage**

| **Tools** | **Total runtime (s)** | **Max memory usage (MB)** |
| --- | --- | --- |
| *dandelionR* | 193.58 | 4293.5 |
| *Dandelion* | 65.74 | 3824.38 |

The runtime of *dandelionR*’s workflow is slightly longer than that of the original *Dandelion* workflow. To further investigate this discrepancy, we divided the pipeline into eight components and profiled the runtime and memory usage of each step individually (**Supplementary Information Table 2)**.

**Supplementary Information Table 2. Breakdown of *dandelionR* sub-functions.**

| **Function** | **Total runtime(s)** | **Max memory usage (MB)** |
| --- | --- | --- |
| *Load data* | 4.23 | 1690.2 |
| *setupVdjPseudobulk* | 6 | 2015.3 |
| *Milo pseudobulk* | 8.45 | 1344.6 |
| *miloUmap* | 178.31 | 1906.8 |
| *VDJ feature space* | 1.4 | 1191.8 |
| *diffusion map* | 3.7 | 1697 |
| *markovProbability* | 11.44 | 1465.1 |
| *project to cells* | 4.14 | 1907.1 |

Note that the memory usage recorded for each individual step reflects only the temporary allocation within its isolated execution. The total peak memory observed during full pipeline execution is larger, due to the cumulative coexistence of intermediate objects in memory.

Among all steps, *miloUmap()* was the primary contributor to the increased runtime. This function performs UMAP on the KNN graph constructed by *MiloR*. Unlike python’s *Scanpy*, which supports direct UMAP on a precomputed KNN graph, R’s *uwot::umap* requires first converting the KNN graph into an adjacency matrix. The matrix-based calculation is substantially more expensive.

To address this inefficiency, we introduced an option to directly run UMAP on the latent representation *X_scvi*. While this approach deviates from *Dandelion*’s original design, the visualization remains qualitatively similar (**Supplementary Information Fig. 2**). After applying this modification, the runtime and memory usage of *miloUmap* step decreased to 25.77 seconds and 350MB, demonstrating a significant speed-up.


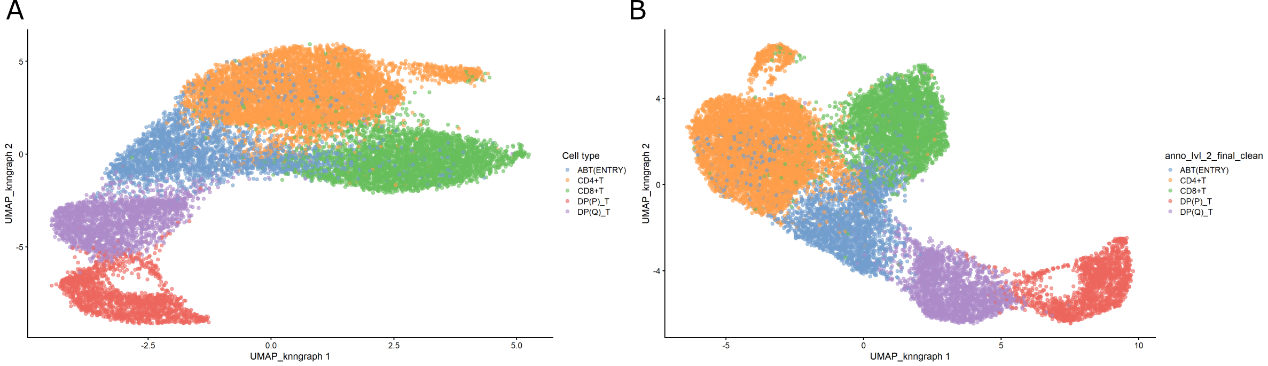


**Supplementary Information Fig. 2 Output of the function *miloUmap*.** (A) UMAP computed from the adjacency matrix (default, graph-based layout). (B) UMAP computed directly from *X_scvi*. While it deviates from *Dandelion*’s original approach, the layout remains visually comparable.

We added a new parameter *`use_graph`* (default: TRUE) to the function *miloUmap()*. When set to FALSE, *dandelionR* will directly compute UMAP on *X_scvi* to accelerate calculation. With *miloUmap(use_graph = FALSE)*, the overall runtime and memory usage of the full *dandelionR* pipeline are reduced to 92.2 seconds and 4107MB, respectively, representing a deviation of 7.39% in runtime and 40.25% in memory compared to original *Dandelion* implementation.
